# Transcriptomic analysis of the effects of Toll-like receptor 4 and its ligands on the gene expression network of hepatic stellate cells

**DOI:** 10.1186/s13069-016-0039-z

**Published:** 2016-02-18

**Authors:** Yangyang Ouyang, Jinsheng Guo, Chenzhao Lin, Jie Lin, Yirong Cao, Yuanqin Zhang, Yujin Wu, Shiyao Chen, Jiyao Wang, Luonan Chen, Scott L. Friedman

**Affiliations:** Division of Digestive Diseases, Department of Internal Medicine, Zhong Shan Hospital, Shanghai Medical College, Fu Dan University, 180 Feng Lin Road, Shanghai, 200032 China; Institutes of Biomedical Sciences, Fu Dan University, Shanghai, 200032 China; Key Laboratory of Systems Biology, Shanghai Institutes for Biological Sciences, Chinese Academy of Sciences, Shanghai, 200032 China; Division of Liver Diseases, Icahn School of Medicine at Mount Sinai, 1425 Madison Ave., Room 11-70C, New York, 10029-6574 NY USA

**Keywords:** Toll-like receptor 4, Hepatic stellate cells, Lipopolysaccharide, High-mobility group box 1, Gene expression network

## Abstract

**Background:**

Intact Toll-like receptor 4 (TLR4) has been identified in hepatic stellate cells (HSCs), the primary fibrogenic cell type in liver. Here, we investigated the impact of TLR4 signaling on the gene expression network of HSCs by comparing the transcriptomic changes between wild-type (JS1) and TLR4 knockout (JS2) murine HSCs in response to two TLR4 ligands, lipopolysacchride (LPS), or high-mobility group box 1 (HMGB1).

**Results:**

Whole mouse genome microarray was performed for gene expression analysis. Gene interaction and co-expression networks were built on the basis of ontology and pathway analysis by Kyoto Encyclopedia of Genes and Genomes (KEGG). Gene expression profiles are markedly different between Wild type (JS1) and TLR4 knockout (JS2) HSCs under basal conditions or following stimulation with LPS or HMGB1. The differentially expressed genes between TLR4 intact and null HSCs were enriched in signaling pathways including p53, mTOR, NOD-like receptor, Jak-STAT, chemokine, focal adhesion with some shared downstream kinases, and transcriptional factors. Venn analysis revealed that TLR4-dependent, LPS-responsive genes were clustered into pathways including Toll-like receptor and PI3K-Akt, whereas TLR4-dependent, HMGB1-responsive genes were clustered into pathways including metabolism and phagosome signaling. Genes differentially expressed that were categorized to be TLR4-dependent and both LPS- and HMGB1-responsive were enriched in cell cycle, ubiquitin mediated proteolysis, and mitogen-activated protein kinase (MAPK) signaling pathways.

**Conclusions:**

TLR4 mediates complex gene expression alterations in HSCs. The affected pathways regulate a wide spectrum of HSC functions, including inflammation, fibrogenesis, and chemotaxis, as well as cell growth and metabolism. There are common and divergent regulatory signaling downstream of LPS and HMGB1 stimulation via TLR4 on HSCs. These findings emphasize the complex cascades downstream of TLR4 in HSCs that could influence their cellular biology and function.

**Electronic supplementary material:**

The online version of this article (doi:10.1186/s13069-016-0039-z) contains supplementary material, which is available to authorized users.

## Background

Hepatic stellate cells (HSCs) are the predominant extracellular matrix-producing cell type in the liver [1–5]. The activation of HSCs is the central event in fibrogenesis that drives fibrosis, cirrhosis, and hepatic decompensation. Following liver injury, the activated HSC adopts a myofibroblast-like phenotype to produce collagen and other extracellular matrix (ECM) components. Activated HSCs also express the intracellular microfilament protein α-smooth muscle actin (α-SMA) and tissue inhibitor of metalloproteinases (e.g., tissue inhibitor of metalloproteinase (TIMP)-1), the latter of which inhibits matrix degradation. The cells acquire chemotactic abilities, which confer upon them the potential to migrate and accumulate. HSCs release profibrogenic and promitogenic cytokines (e.g., TGFβ1 and PDGF), which stimulate ECM production and drive proliferation in an autocrine manner [6]. Activated HSCs are resistant to apoptotic stimuli and express pattern recognition receptors, specifically Toll-like receptors 4 (TLR4) and 9 (TLR9), and respond to their ligands to activate downstream signalling pathways and transcriptional factors (TFs).

TLR4 is a member of the pattern recognition receptor superfamily. It plays an important role in recognizing bacterial lipopolysaccharide (LPS) and mediating inflammatory responses and innate immunity [7, 8]. TLR4 signals through the adaptor protein MyD88 in activating downstream effectors that include nuclear factor-κB (NF-κB), mitogen-activated protein kinase (MAPKs), and phosphatidylinositol 3-kinase (PI3K), leading to the production of pro-inflammatory cytokines. The MyD88-independent pathway is associated with the induction of IFN-β- and IFN-inducible genes. In addition to its exogenous ligand, LPS, there are endogenous TLR4 ligands from cellular compartments that are increased during tissue injury. Notably, high-mobility group box 1 (HMGB1), a chromatin associated highly conserved nuclear protein, may serve as an extracellular signaling molecule and damage associated molecular pattern molecule (DAMP) that activates Toll-like receptor signaling [9, 10]. HMGB1 is passively released from necrotic cells and is actively secreted by inflammatory cells, mediating the response to inflammation, immunity, chemotaxis, and tissue regeneration [11–18]. The level of HMGB1 is increased in the serum of chronic hepatitis patients [19] and in the livers of experimental liver fibrosis [20]. Increased levels of HMGB1 are closely associated with the severity of inflammation and fibrosis [20].

HSCs have intact TLR4 signaling. They express LPS-recognizing receptors including CD14, TLR4, and MD2 and respond to LPS with the activation of iκB kinase (IKK)/NF-κB and JNK, as well as the secretion of pro-inflammatory cytokines (e.g., IL-6 and TNF-α), chemokines (e.g., MCP-1, MIP-2, RANTES, and CCR5), and expression of adhesion molecules (ICAM-1) [21]. TLR4 signaling contributes to the activation of hepatic stellate cells (HSC) by promoting an inflammatory phenotype, fibrogenesis, and cell survival [22]. In culture, this cell type responds to HMGB1 via TLR4, which subsequently triggers inflammation and enhances fibrogenic responses via downstream signaling [23], indicating that TLR4 signaling need not rely solely on gut-derived LPS for activation during liver injury.

In this study, we have explored the broad impact of TLR4 signaling on HSC gene expression and signaling pathways and the common and differential effects of the TLR4 activation by LPS or HMGB1, which represent the exogenous and endogenous ligands of TLR4, respectively. Through this effort, we seek to identify both the common and differentially expressed genes of HSC in response to different TLR4 ligands and to uncover the key regulatory molecules.

## Results

### Comparison of the transcriptome of JS1 and JS2 cells

The transcriptomic changes within wild-type (JS1) and TLR4 knockout (JS2) mouse stellate cell lines were investigated. The gene expression patterns of JS1 and JS2 cells were significantly different under basal conditions. The genes that were 1.5-fold differentially expressed and the numbers of Go terms that were enriched by these genes are listed in Additional file 1: Table S1. The differentially expressed genes included those linked to fibrogenesis (Col I, Col III, FN1), matrix remodeling (TIMP2, TIMP3, MMP2), growth factors and their receptors (VEGFD, FGF7, IGF1 and IGF1R, PDGFα and PDFGRα、PDGFR), chemokine and chemokine receptors (CXCL12, CXCL11, of CXCR7), inflammation and immune mediators (IL6), transcription factors and some important signaling molecules (Jun, Stat3, MAPK1). The expression of these genes was validated by qRT-PCR. A high correlation was observed between q-PCR and microarray data (Table 1).

**Table 1:** Verification of gene expression changes by RT-qPCR

Gene symbol	Gene name	Primer sequences	Expression of fold change (log2)*		Gene function
			Gene Chip	q-PCR	
SP1	Specific protein 1	F:5′-ACTGAGATCCCCAAAACACC-3′; R:5′-TTCTCTGCCCTCACTCTTGA-3′	2.00	3.81	Transcriptional factor
STAT3	Signal transducer and activator of transcription 3	F:5′-TCACTTGGGTGGAAAAGGAC-3′; R:GGAATGTCGGGGTAGAGGTAG-3′	1.89	2.35	Transcriptional factor
Jun	Jun proto-oncogene	F:5′-CCTTCTACGACGATGCCCTC-3′; R:5′-GGTTCAAGGTCATGCTCTGTTT-3′	3.01	3.94	Transcriptional factor
Fas	Fas cell surface death receptor	F:5′-GCAGACATGCTGTGGATCTGG-3′; R:5′-TCACAGCCAGGAGAATCGCAG-3′	5.10	2.18	Transcriptional factor
FGF7	Fibroblast growth factor 7 (keratinocyte growth factor)	F:5′-ACGGCTACGAGTGTGAACTGT-3′; R:5′-TTTCACTTTGCCTCGTTTGTC-3′	75.96	42	Growth factor
BDNF	Brain-derived neurotrophic factor	F:5′-GCCTCCTCTACTCTTTCTGCTG-3′; R:5′-TGTGACCCACTCGCTAATACTG-3′	2.68	5.76	Growth factor
VEGFD (FIGF)	c-fos induced growth factor (vascular endothelial growth factor D)	F:5′-AGCACCTCCTACATCTCCAAAC-3′; R:5′-CATTCATCTTCTTCTGGGGTCT-3′	4.82	5.76	Growth factor
IGF1	Insulin-like growth factor 1	F:5′-AAAATCAGCAGCCTTCCAACT-3′; R:5′-CCTGTGGGCTTGTTGAAGTAA-3′	2.17	3.13	Growth factor
PTN	Pleiotrophin	F:5′-CTCTGCACAATGCTGACTGTC-3′; R:5′-CTTTGACTCCGCTTGAGGCTT-3′	31.78	36	Growth factor
PDGFα	Platelet-derived growth factor, alpha	F:5′-TGGCTCGAAGTCAGATCCACA-3′; R:5′-TTCTCGGGCACATGGTTAATG-3′	1.61	1.229	Growth factor
IGFBP3	Insulin-like growth factor binding protein 3	F:5′-TCCAGGAAACATCAGTGAGTCCGA-3′; R:5′-CATACTTGTCCACACACCAGCAGA-3′	22.75	27.84	Insulin-like growth factor binding protein
Col I	collagen, type I	F:5′-GCTCCTCTTAGGGGCCACT-3′; R:5′-CCACGTCTCACCATTGGGG-3′	3.56	1.6	Fibrogenesis
FN1	Fibronectin 1	F:5′-TTCAAGTGTGATCCCCATGAAG-3′; R:5′-CAGGTCTACGGCAGTTGTCA-3′	7.34	5.26	Fibrogenesis
TIMP2	Tissue inhibitors of metalloproteinase 2	F:5′-CTGGACGTTGGAGGAAAGAAG-3′; R:5′-CTGGGTGATGCTAAGCGTGTC-3′	2.01	3.456	Matrix remodeling
TIMP3	Tissue inhibitors of metalloproteinase 3	F:5′-GCAAGGGCCTCAATTACCG-3′; R: 5′-AGGCGTAGTGTTTGGACTGATA-3′	8.57	10.389	Matrix remodeling
MMP2	Matrix metalloproteinase 2	F:5′-GTGTCTTCCCCTTCACTTTCCT-3′; R:5′-CATCATCGTAGTTGGTTGTGGT-3′	8.07	10.24	Matrix remodeling
IL6	Interleukin 6	F:5′-GGAGAGGAGACTTCACAGAGGA-3′; R:5′-ATTTCCACGATTTCCCAGAGA-3′	3.00	1.74	Inflammatory factor
CXCR7	Chemokine (C-X-C motif) receptor 7	F:5′-AGCCTGGCAACTACTCTGACA-3′; R:5′-GAAGCACGTTCTTGTTAGGCA-3′	11.31	14.42	Chemokine receptor
CXCL11	Chemokine (C-X-C motif) ligand 11	F:5′-GGCTTCCTTATGTTCAAACAGGG-3′; R:5′-GCCGTTACTCGGGTAAATTACA-3′	2.03	0.65	Chemokine
CXCL12	Chemokine (C-X-C motif) ligand 12	F:5′-ACTGTGCCCTTCAGATTGTTG-3′; R:5′-CAGCCTTTCTCTTCTTCTGTCG-3′	44.84	34.95	Chemokine
CASP2	Caspase 2	F:5′-GCAAGATGGAAAGAACCACAC-3′; R:5′-GCAAGATGGAAAGAACCACAC-3′	1.83	1.7	Apoptin
DLK1	Delta-like 1 homolog (Drosophila)	F:5′-AGTGCGAAACCTGGGTGTC-3′; R:5′-GCCTCCTTGTTGAAAGTGGTCA-3′	18.95	19.784	Tumor repressor, cell differentiation
AKT3	Thymoma viral proto-oncogene homolog 3	F:5′-GTGGACTTACCTTATCCCCTCA-3′; R:5′-TTGGCTTTGGTCGTTCTGTTT-3′	2.50	3.27	Serine/threonine protein kinase
GNG2	Guanine nucleotide binding protein (G protein), gamma 2	F:5′-GAAGCCAACATCGACAGGAT-3′; R:5′-GTTTTCTGAGGCTGGGACTG-3′	5.71	5.5	Signaling transduction
MDM2	E3 ubiquitin protein ligase	F:5′-AGATCCTGAGATTTCCTTAGCTGACT-3′; R:5′-TCTCACGAAGGGTCCAGCATCT-3′	3.77	4	Ubiquitination
Wnt5a	Wingless-related MMTV integration site 5A	F:5′-CAACTGGCAGGACTTTCTCAA-3′; R:5′-CATCTCCGATGCCGGAACT-3′	2.35	3.73	Modulator of Wnt signaling

*P < 0.05, gene mRNA expression in JS1 cells when compared to JS2

Pathway analysis was used to uncover the significant pathways within differentially expressed gene sets according to the Kyoto Encyclopedia of Genes and Genomes (KEGG) database. Of the data set of differentially expressed genes in JS1 compared to JS2, 682 up-regulated genes and 773 down-regulated genes populated 17 up- and 10 down-pathway categories, respectively. Seven of the signaling pathways up-regulated in JS1 cells and correlated with key HSC functions were p53, mTOR, NOD-like receptor, Jak-STAT, chemokine, focal adhesion, and pathways in cancer (Table 2). Important down-regulated signaling pathways included those regulating cell adhesion molecules (CAMs), phagosome activity, axon guidance, and antigen processing and presentation (Table 2).

**Table 2:** KEGG pathway analysis for differentially expressed genes between JS1 and JS2 cells and the key regulatory genes

Pathway ID	Pathway term	Enrichment (+, up; −, down)	*P* value	No. of DifGenes	Gene-act-network core genes (degree >5)	Co-expression network core genes (Dif-degree >5)
path:mmu04115	p53 signaling pathway	+1.971	0.013637	18	Ccnd1, Igf1	Apaf1
path:mmu0415	mTOR signaling pathway	+1.934	0.030489	14	***Rps6ka1***, Pik3r3, Mapk1, Igf1, Braf, Ins2	***Rps6ka1***
path:mmu04621	NOD-like receptor signaling pathway	+1.857	0.0298641	16	Mapk1, Mapk12, Map3k7	Tab3, Birc2
path:mmu04630	Jak-STAT signaling pathway	+1.511	0.049774	25	***Jak2***,***Stat5b***,***Stat5a***, ***Il6st***, Pik3r3, Akt3,Ptpn11,Stat3,Ifnar1,Stat6, Stat2	***Jak2***, ***Stat5a, Stat5b***, ***Il6st,*** Grb2***,*** Il20rb, Socs5
path:mmu04062	Chemokine signaling pathway	+1.489	0.042555	29	***Jak2***,***Prkacb***, ***Gsk3b,*** Mapk1, Kras, Plcb3, Gnai3, Cdc42, Braf,Stat5b, Stat3, Shc4, Pard3, Il11ra1	***Jak2***, ***Prkacb*** **,** ***Gsk3b***, Grb2
path:mmu04510	Focal adhesion	+1.469	0.025892	39	***Pdgfra***, ***Pdgfrb***, ***Prkca***, ***Gsk3b***, ***Fn1,*** Mapk1, Itga11, Igf1r, Jun,Akt3, Igf1, Tnc, Braf, Col5a1, Col3a1, Col1a2, Col1a1, Thbs3, Thbs1, Col6a2, Col6a1, Col4a6, Tln2, Ugdh	***Pdgfra***, ***Pdgfrb, Prkca***, ***Gsk3b, Fn1***, Grb2, Birc2, Ppp1cb
path:mmu05200	Pathways in cancer	+1.350	0.032167	57	***Pdgfra***, ***Pdgfrb***, ***Prkca***, ***Grb2***, ***Gsk3b***, ***Fn1,*** Pik3r3, Egfr, Mapk1, Kras, Jun, Akt3, Igf1, Braf, Stat5b, Stat5a, Figf, Cdk4, Stat3, Pias1, Mdm2	***Pdgfra***, ***Pdgfrb, Prkca***, ***Gsk3b***, ***Fn1,*** Mmp2, Flt3l, Birc2, Brca2, Appl1, Casp3, Fzd1, Msh2
path:mmu04514	Cell adhesion molecules (CAMs)	−1.493	0.046196	28	***H2-M2***, ***LOC547349***, ***H2-Q7, H2-Q2***, ***Itgb7***, ***Sdc3,*** LOC100044874, H2-M3, H2-T23, H2-T10, H2-K1, H2-Q8,H2-Q6, H2-Q10, Itgb6, H2-D1, Sdc2	***H2-M2***, ***H2-Q7, Itgb7***, ***LOC547349***, ***H2-Q2***, ***Sdc3,*** F11r, Madcam1, Selp
path:mmu04145	Phagosome	−1.640	0.006056	41	***H2-M2***, ***H2-Q2, H2-Q7, LOC547349***, LOC100044874, H2-M3, H2-T23, H2-T10, H2-K1, H2-Q8, H2-Q6, H2-Q10, H2-D1, Itgb5, Itgb3, Itga5, Actg1	***H2-M2***, ***H2-Q2, H2-Q7***, ***LOC547349***, Atp6v0b, Atp6v1h, Atp6v1e1, Sec61b, Atp6v0c
path:mmu04360	Axon guidance	−1.653	0.013723	33	***Hras1,*** Mapk3, Ptk2, Nras, Tapbp	***Hras1,*** Sema3a, Sema4f, Ephb3, Nfatc2
path:mmu04612	Antigen processing and presentation	−1.886	0.012941	21	***H2-M2, H2-Q2***, ***H2-Q7***, ***LOC547349,*** LOC100044874, H2-M3, H2-T23, H2-T10, H2-K1, H2-Q6, H2-Q8, H2-Q10, H2-D1, Tapbp	***H2-M2***, ***H2-Q2, H2-Q7*** **,** ***LOC547349***

Note: Genes that are identified to be key regulatory factors by both gene-act-net work and co-expression network analysis (degree or difference degree >5) are marked in bold and italic font

**Fig. 1:**
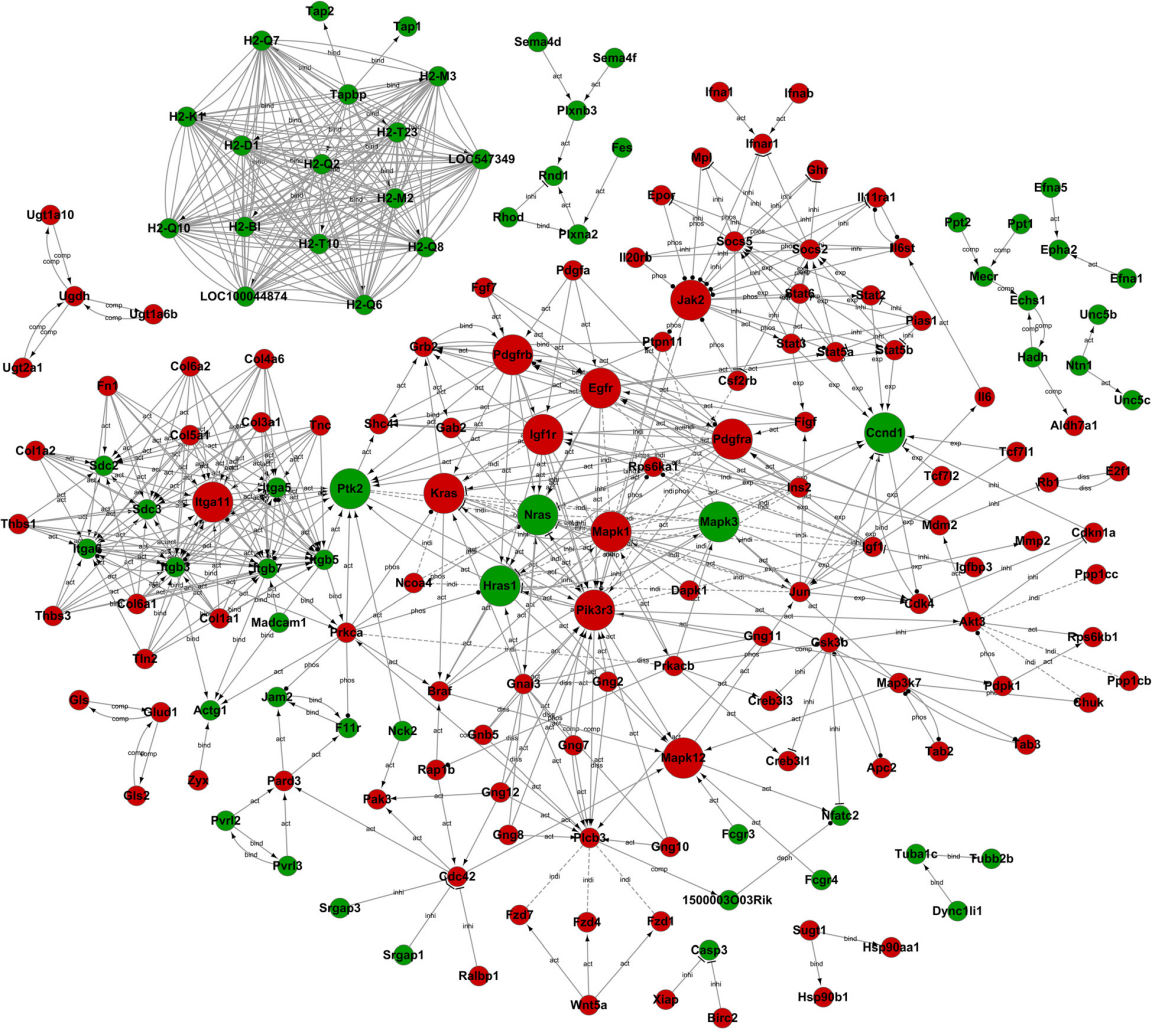
Gene-act-network analysis of the effect of TLR4 on the biological functions of hepatic stellate cells. The networks were built according to the relationship between the differentially expressed genes in TLR4 intact and null HSC using the KEGG database. *Green circles* represented down-regulated genes; *red circles* represent the up regulated genes; → activation/association; —: compound; —|: inhibition. The sizes of the circles were correlated to the degree numbers linked to the genes

To illustrate the effect of TLR4 on biological functions of HSCs, we built the gene-act-network according to the relationship between the differentially expressed genes in TLR4 intact and null HSC using the KEGG database (Fig. 1). Protein complexes and functional modules were distinguished which have different biological implications. In this network of gene–gene interaction, the genes that were the central regulatory factors due to a strong degree of centrality (degree >5) were listed in Table 2. Specifically, Ccnd1, Igf1, MAPK family members (MAPK1, MAPK12, MAP3K7), Rps6ka1, Pik3r3, Jak2, Stat5, Stat3, PDGFRα and β, Prkca, Gsk3b, Fn1, Itgβ7, JUN, H-ras, and multiple histocompatibility complex molecules (H2-M2, H2-Q7, H2-Q2, LOC547349) were involved in the previously mentioned pathways.

By co-expression network analysis, four networks were identified in JS1 cells using differentially expressed genes populating the pathways category. There were 172 genes related to one another. Likewise, three networks were identified in JS2 cells (Additional file 2: Figure S1). Core regulatory factors that involved in the differential networks were determined by the degree differences between the JS2 and JS1 cells. Genes that displayed degree differences more than 5 are shown in Table 2. Of note, some of the genes identified to be core regulatory factors in co-expression networks were also the central regulatory factors in gene-act-network, indicating their important role in the differentially expressed gene spectrums in JS1 and JS2 cells. These included Rps6ka1, Jak2, Stat5, Il6st, Prkca, Fn1, GSk3b, PDGFRα and β, Itgβ7, and multiple histocompatibility complex molecules (H2-M2, H2-Q7, H2-Q2, LOC547349) and are highlighted in Table 2.

### Comparison of the transcriptome between JS1 and JS2 cells in response to LPS

A total of 1392 different probes were tested with 849 up-regulated and 543 down-regulated in JS1 cells in response to LPS stimulation. Gene ontology analysis indicated that 69 up and 86 down gene ontology (GO) terms were enriched (Additional file 1: Table S1). Pathway analysis identified 261 up-regulated genes and 140 down-regulated genes populated 10 up and 8 down pathways categories, respectively. The signaling pathways up-regulated in LPS-treated JS1 cells included Toll-like receptor, neurotrophin signaling, glycolysis/gluconeogenesis, and immune disease pathways (Table 3), with MAPKs (MAPK9, MAPK14) and multiple MHC molecule (H2-Q2); these were the core regulatory factors in gene-act-net work (Additional file 3: Figure S2) and co-expression network (Additional file 4: Figure S3), with the highest degree and differential degree numbers. The down-regulated signaling pathways included phosphatidylinositol signaling, tight junction, and ubiquitin-mediated proteolysis, with Prkca, Map3k1, and Herc1 as the core regulatory factors (Table 3). The gene interaction and co-expression networks in TLR4 null cells post LPS stimulation were significantly simpler and lacked core regulatory factors (Additional file 3: Figure S2).

**Table 3:** KEGG pathway analysis for differentially expressed genes in JS1 cells with or without LPS treatments and the key regulatory genes

Path	Path Term	Enrichment (+, up; −, down)	*P* value	No. of genes	Gene-act-network core genes (degree >3)	Co-expression network core genes (Dif-degree >3)
path:mmu00010	Glycolysis/gluconeogenesis	+3.226	0.002679	10	Hk1	Gm5506, Tpi1, Ldha
path:mmu03420	Nucleotide excision repair	+3.110	0.008222	8	/	Rfc2, Ercc1, Cdk7
path:mmu03040	Spliceosome	+2.030	0.013765	15	/	Ddx39b, Snrpb, Rpa2, Prpf19, Ppie, Hnrnpa3
path:mmu04620	Toll-like receptor signaling pathway	+2.224	0.018448	11	***Mapk9***, ***Mapk14***	***Mapk9***, ***Mapk14***
path:mmu04722	Neurotrophin signaling pathway	+1.965	0.021031	14	***Mapk14,*** Cdc42	***Mapk14***
path:mmu05320	Autoimmune thyroid disease	+2.589	0.028615	7	***H2-Q2,*** H2-Q10,LOC100044874,H2-T10,H2-Bl	***H2-Q2***
path:mmu03030	DNA replication	+2.757	0.032638	6	/	Pola2
path:mmu04070	Phosphatidylinositol signaling system	−3.880	0.003976	7	***Prkca,*** Pik3r3	***Prkca***
path:mmu04530	Tight junction	−2.431	0.025024	8	***Prkca***, Myl9	/
path:mmu04120	Ubiquitin mediated proteolysis	−2.231	0.028651	9	***Map3k1***	***Map3k1,*** Herc1, Prkca, Ubr5

Note: Genes that are identified to be key regulatory factors by both gene-act-network and co-expression network analysis (degree or difference degree >3) are marked in bold and italic font

### Comparison of the transcriptome of JS1 and JS2 cells in response to HMGB1

A total of 1445 different probes were tested with 586 up-regulated and 859 down-regulated transcripts in JS1 cells in response to HMGB1 stimulation. Gene ontology analysis indicated that 47 up and 95 down GO terms were enriched (Additional file 1: Table S1). Pathway analysis identified 184 up-regulated genes and 219 down-regulated genes populated 8 up- and 5 down-pathways categories, respectively. Within the signaling pathways up-regulated in HMGB1-treated JS1 cells, there were glutathione metabolism and drug metabolism—cytochrome P450 (Table 4), with Gpx4, Gstt2, and Odc1. Cyp2e1 as the core regulatory factors in gene act network (Additional file 5: Figure S4) and/or co-expression network (Additional file 6: Figure S5), with highest degree and differential degree numbers. Within the down-regulated signaling pathways, there were ubiquitin-mediated proteolysis, mTOR signaling, pathways in cancer, RIG-like receptor signaling pathway with Herc1 and Traf6, Mapk1 and Rps6ka3, Ifg1r, Prkx, and Foxo1 as the core regulatory factors (Table 4).

**Table 4:** KEGG pathway analysis for differentially expressed genes in JS1 cells with or without HMGB1 treatments and the key regulatory genes

Path	Path term	Enrichment (+, up; −, down)	*P* value	No. of genes	Gene-act-network core genes (degree >3)	Co-expression network Dif-degree >3
path:mmu03410	Base excision repair	+4.857	0.001406	7	/	Nthl1, Mpg
path:mmu00480	Glutathione metabolism	+3.001	0.022114	6	***Gpx4***,***Gstt2***,Gstp1,Mgst3,Gstp2	***Gstt2***, ***Gpx4, Odc1***
path:mmu00982	Drug metabolism—cytochrome P450	+2.805	0.028716	6	***Gstt2***,***Cyp2e1,*** Mgst3,Gstp1	***Gstt2, Cyp2e1***
path:mmu04120	Ubiquitin-mediated proteolysis	−2.378	0.003899	15	Traf6	Ube2n, Uba3, Herc1
path:mmu04150	mTOR signaling pathway	−3.012	0.014331	7	Mapk1	Rps6ka3
path:mmu05200	Pathways in cancer	−1.622	0.030471	22	Igf1r,Prkca,Mapk1,Ptk2	Rala, E2f3, Foxo1
path:mmu04622	RIG-I-like receptor signaling pathway	−2.342	0.041959	7	Traf6	Cyld

Note: Genes that are identified to be key regulatory factors by both gene-act-network analysis and co-expression network analysis (degree or difference degree >3) are marked in bold and italic font

Similar to the response to LPS, the gene interaction and co-expression networks in TLR4 null cells following HMGB1 stimulation were also significantly simpler and lacked core regulatory factors (Additional file 5: Figure S4 and Additional file 6: Figure S5).

### Venn analysis of TLR4-dependent LPS and HMGB1 response in JS1 cells

**Fig. 2:**
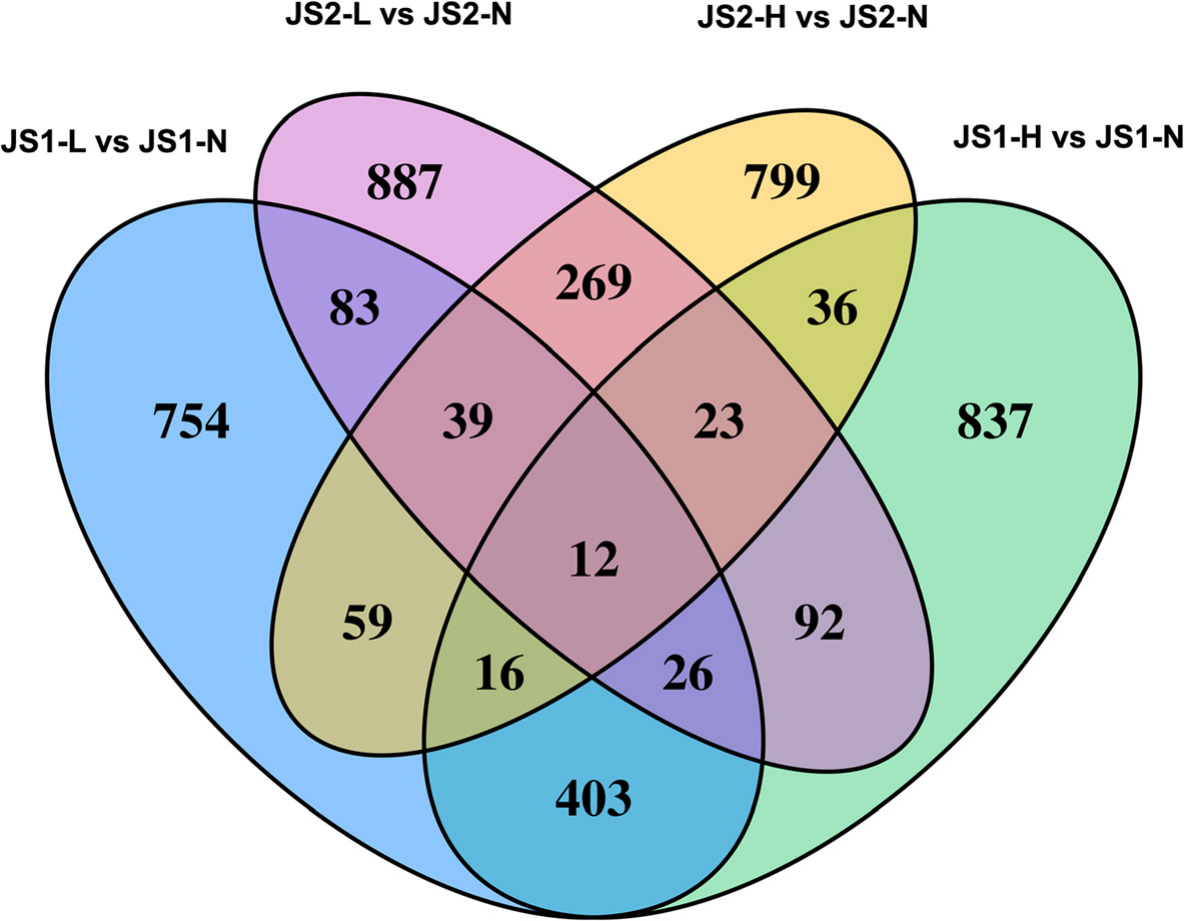
Venn analysis of the common and divergent TLR4-dependent genes expressed in response to LPS or HMGB1 stimuli. *1Lvs1N:* number of differentially expressed genes in JS1 cells in response to LPS stimulation, *2Lvs2N:* number of differentially expressed genes in JS2 cells in response to LPS stimulation, *1Hvs1N:* number of differentially expressed genes in JS1 cells in response to HMGB1 stimulation, *2Hvs2N:* number of differentially expressed genes in JS2 cells in response to HMGB1 stimulation. The overlaps represent the common differentially expressed genes in both treatments and/or cells

In order to compare the common and differential TLR4-dependent responses of JS1 cells to LPS and HMGB1, we further performed Venn analysis to identify the common and specific transcriptomic responses and the gene interactions of HSCs in response to LPS or HMGB1 via TLR4 (Fig. 2).

**Fig. 3:**
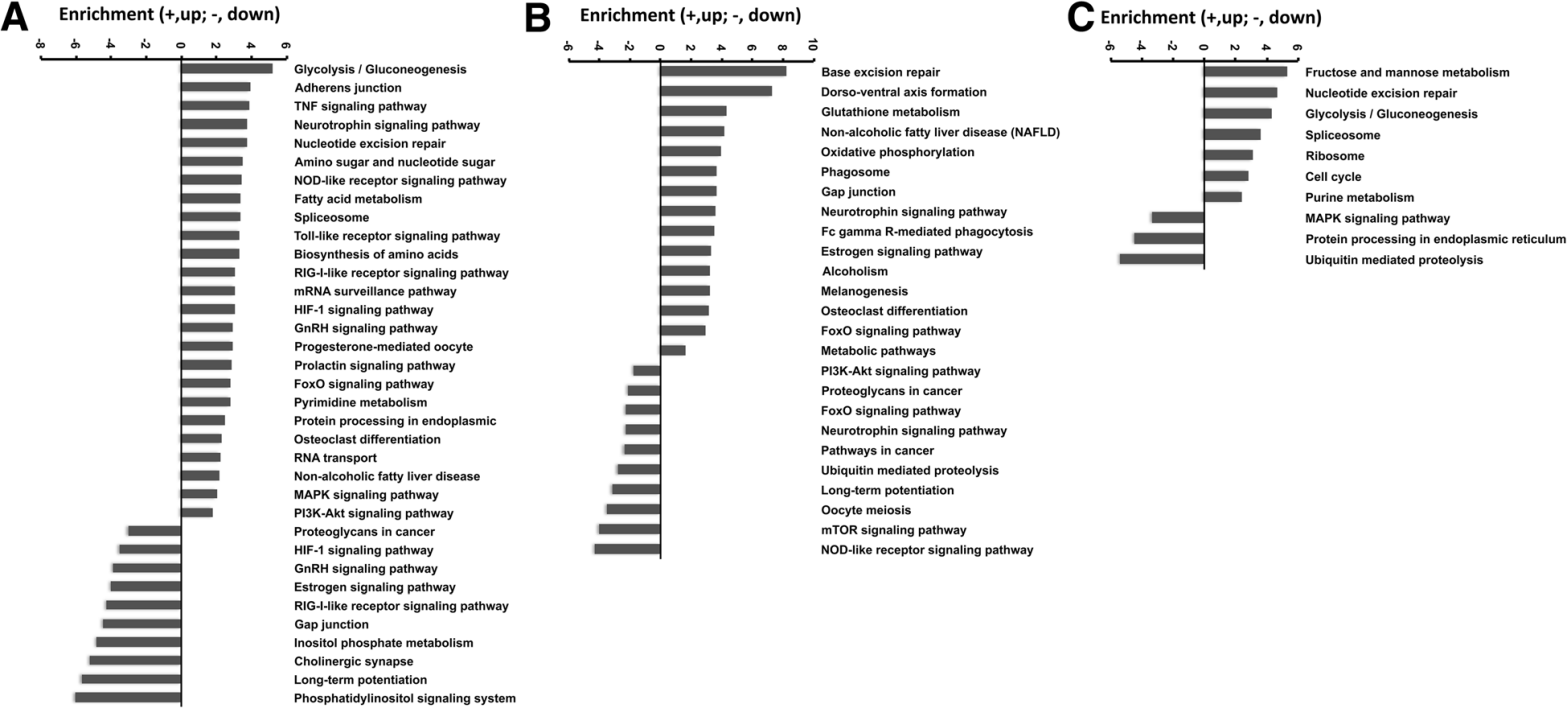
Pathway analysis for the common and divergent TLR4-dependent genes expressed in response to LPS or HMGB1 stimuli. **A**: The significant pathways of the differentially expressed genes that were LPS responsive only in JS1 cells vs JS2 cells. **B**: The significant pathways of the differentially expressed genes that were HMGB1responsive only in JS1 cells vs JS2 cells. **C**: The significant pathways of the differentially expressed genes that were both LPS and HMGB1 responsive in JS1 cells vs JS2 cells. *P* value < 0.05 and FDR < 0.05 were used as a threshold to select significant KEGG pathways. *Y* axis, enrichment of the significant pathway; *X* axis, KEGG pathway term

**Fig. 4:**
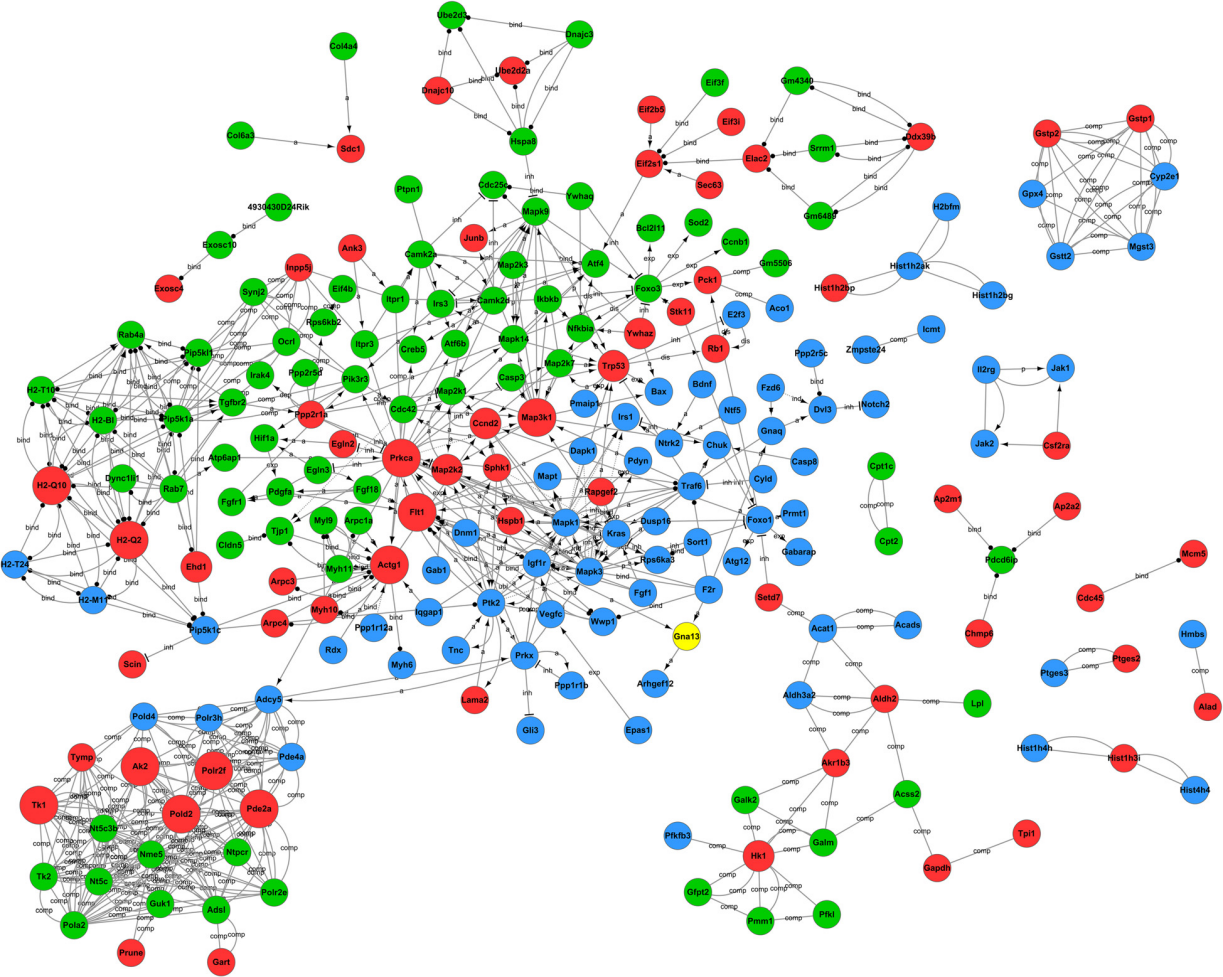
Venn analysis to identify the common and specific transcriptomic responses and the gene interaction of HSCs to LPS or HMGB1 via TLR4. *Green circles* represented differentially expressed genes belong to LPS responsive only in JS1 cells vs JS2 cells. *Blue circles* represented differentially expressed genes belong to HMGB1 responsive only in JS1 cells vs JS2 cells. *Red circles* represented the differentially expressed genes belong to both LPS and HMGB1 responsive in JS1 cells vs JS2 cells

Seven hundred fifty-four differentially expressed genes were categorized to be TLR4-dependent and LPS-specific responses. Among them, 179 up-regulated genes were enriched into 25 up-regulated pathways including Toll-like receptor, neurotrophin, MAPK, PI3K-Akt, TNF, Foxo, and osteoclast differentiation (Fig. 3a), with Mapk9, Mapk14, Map2k1, and Foxo3 as the core regulatory factors; on the other hand, 77 down-regulated genes were enriched into 20 down-regulated pathways including phosphatidylinositol signaling system, with Pik3r3 as a core regulatory factor (Table 5, Fig. 4).

**Table 5:** Venn-analysis for pathways of differentially expressed genes that were LPS responsive only in JS1 cells vs JS2 cells and the key regulatory genes

Pathway ID	Pathway term	Enrichment	*P* value	No. of DifGenes	Gene-act-net
		(+, up; −, down)			core genes (degree >5)
PATH:04668	TNF signaling pathway	3.8644	0.0005	10	Mapk9, Mapk14, Map2k1
PATH:04722	Neurotrophin signaling pathway	3.7442	0.0002	12	Mapk9, Foxo3, Mapk14, Map2k1
PATH:04621	NOD-like receptor signaling pathway	3.4526	0.0195	5	Mapk9, Mapk14
PATH:04620	Toll-like receptor signaling pathway	3.3364	0.0044	8	Mapk9, Mapk14, Map2k1
PATH:04622	RIG-I-like receptor signaling pathway	3.0523	0.0301	5	Mapk9, Mapk14
PATH:04066	HIF-1 signaling pathway	3.03588	0.0074	8	Map2k1
PATH:04068	FoxO signaling pathway	2.8081	0.0075	9	Mapk9, Foxo3, Mapk14, Map2k1
PATH:04141	Protein processing in endoplasmic reticulum	2.4924	0.0106	10	Mapk9
PATH:04380	Osteoclast differentiation	2.3217	0.0396	7	Mapk9, Mapk14, Map2k1
PATH:04010	MAPK signaling pathway	2.0206	0.0189	13	Mapk9, Mapk14, Map2k1
PATH:04151	PI3K-Akt signaling pathway	1.8052	0.0291	15	Foxo3, Map2k1
PATH:04066	HIF-1 signaling pathway	-3.5287	0.0321	4	Pik3r3
PATH:04070	Phosphatidylinositol signaling system	-6.0445	0.0021	5	Ocrl, Pik3r3, Synj2

Eight hundred thirty-seven differentially expressed genes were found to be TLR4-dependent and HMGB1-specific responses. Within them, 94 up-regulated genes were enriched into 27 up-regulated pathways including glutathione metabolism, metabolic, neurotrophin, osteoclast differentiation, and phagosome signaling (Fig. 3b), with the core regulatory molecules including Gstt2, Mgst3, Cyp2e1, MAPK3, Adcy5, Kras, H2-M11, and H2-T24. One hundred seventy-three down-regulated genes were enriched into 25 down-regulated pathways including Foxo, long-term potentiation, mTOR, neurotrophin, NOD-like receptor, PI3K-Akt, and ubiquitin-mediated proteolysis signaling pathways, with MAPK1, Traf6, Prkx, Igflr, Ptk2, Rps6k3, and Foxo1 as the core regulatory factors (Table 6, Fig. 4).

**Table 6:** Venn analysis for pathways of differentially expressed genes belong to HMGB1 treatment only in JS1 cells vs JS2 cells, and the key regulatory genes

Pathway ID	Pathway term	Enrichment (+, up; −, down)	*P* value	No. of DifGenes	Gene-act-net core genes (degree >5)
PATH:00480	Glutathione metabolism	4.2971	0.0380	3	Gstt2, Mgst3
PATH:04145	Phagosome	3.6668	0.0026	8	H2-M11, H2-T24
PATH:04722	Neurotrophin signaling pathway	3.5650	0.0094	6	Mapk3, Kras, Irs1
PATH:04380	Osteoclast differentiation	3.1579	0.0264	5	Mapk3
PATH:04068	FoxO signaling pathway	2.9708	0.0327	5	Mapk3, Kras, Irs1
PATH:01100	Metabolic pathways	1.6410	0.0234	25	Pold4, Polr3h, Cyp2e1, Pip5k1c
PATH:04151	PI3K-Akt signaling pathway	−1.7433	0.0427	14	Mapk1, Igf1r, Ptk2, F2r, Irs1, Chuk
PATH:05205	Proteoglycans in cancer	−2.1213	0.0215	11	Mapk1, Igf1r, Ptk2, Prkx
PATH:04068	FoxO signaling pathway	−2.2599	0.0443	7	Mapk1, Igf1r, Foxo1, Irs1, Chuk
PATH:04722	Neurotrophin signaling pathway	−2.2599	0.0443	7	Mapk1, Traf6, Irs1, Rps6ka3
PATH:05200	Pathways in cancer	−2.3845	0.0014	18	Mapk1, Igf1r, Ptk2, Traf6, Foxo1, Chuk
PATH:04120	Ubiquitin mediated proteolysis	−2.8018	0.0075	9	Traf6, Wwp1
PATH:04720	Long-term potentiation	−3.1582	0.0267	5	Mapk1, Prkx, Rps6ka3
PATH:04150	mTOR signaling pathway	−4.0231	0.0056	6	Mapk1, Irs1, Rps6ka3
PATH:04621	NOD-like receptor signaling pathway	−4.2869	0.0042	6	Mapk1, Traf6, Chuk'

By Venn analysis, 403 differentially expressed genes were clustered as TLR4-dependent and both LPS and HMGB1 responsive; within them, 107 up-regulated genes were enriched in 9 up-regulated pathways including cell cycle, spliceosome, ribosome, glycolysis/gluconeogenesis, fructose and mannose metabolism, and purine metabolism (Fig. 3c), with TRP53, Ccnd2, HK1, Ddx39b, and Ak2 as the core regulatory genes. In addition, 50 down-regulated genes enriched to 5 down-regulated pathways, which included ubiquitin-mediated proteolysis, protein processing in endoplasmic reticulum, and MAPK signaling pathways with Herc1 and 4, Eif2s1, and Prkca and Map3k1 as the core regulatory genes (Table 7, Fig. 4).

**Table 7:** Venn analysis for pathways of differentially expressed genes belong to both LPS and HMGB1 treatment in JS1 cells vs JS2 cells, and the key regulatory genes

Pathway ID	Pathway term	Enrichment (+, up; −, down)	*P* value	No. of DifGenes	Gene-act-net core genes (degree >5)
PATH:03420	Nucleotide excision repair	4.6978	0.0308	3	Pold2
PATH:00010	Glycolysis/gluconeogenesis	4.3364	0.0171	4	Hk1
PATH:03040	Spliceosome	3.5744	0.0052	7	Ddx39b
PATH:04110	Cell cycle	2.7963	0.0403	5	TRP53, Ccnd2
PATH:00230	Purine metabolism	2.4022	0.0476	6	Pde2a, Polr2f, Ak2
PATH:04010	MAPK signaling pathway	−3.3387	0.0133	6	Prkca, Map3k1, Hspb1
PATH:04141	Protein processing in endoplasmic reticulum	−4.4615	0.0075	5	Eif2s1
PATH:04120	Ubiquitin-mediated proteolysis	−5.3857	0.0035	5	Map3k1

## Discussion

In our previous study, immortalized mouse stellate cell lines that were TLR4 wild-type (JS1) and TLR4 knockout (^−/−^) (JS2) were generated as a useful tool to further delineate the functional role of TLR4 in HSCs [22]. JS2 cells were characterized by lack of LPS responsiveness with lower NF-κB activation and pro-inflammatory cytokine expression than JS1 cells. The TLR4 null cells also showed reduced cell growth and lowered apoptotic threshold following apoptotic stress. By comparing the transcriptomes of these cell lines in the present study, it is clear that TLR4 is critical in maintaining the gene-act-network of HSCs, linking many important cellular signaling pathways, including focal adhesion, p53, NOD-like receptor, mTOR, chemokine, and Jak-STAT. All these signaling pathways have been identified in HSCs and have vital activities that are correlated to cell growth and survival, fibrogenic function, inflammatory phenotype, and immune regulation [24–34]. By gene-act-network analysis and/or co-expression network analysis, some of the signaling pathways shared key regulatory genes, for example, MAPK for focal adhesion, NOD-like receptor, mTOR, pathway in cancer signaling pathways, and transcriptional factor Jun for focal adhesion and pathways in cancer, STAT5 for JAK-STAT and chemokine signaling pathways. Based on these findings, loss of TLR4 in HSCs would attenuate the activities of other signaling pathways that share common downstream kinases and transcription factors.

The transcriptomic analysis also revealed that there were down-regulated genes in JS1 cells compared to JS2 cells, which were enriched within pathways of CAMs, phagosome, axon guidance, and antigen processing and presentation, with MHC I molecule LOC547349 and MHC II molecules including H2-M2, H2-Q7, and H2-Q2 as the key regulatory genes. Future studies should be performed to determine the exact impact of TLR4 on the antigen processing and immune inhibitory function of HSCs [35–38]. In addition, the present study also identified the co-expression of genes (e.g., Sema3a; Sema4f, Ephb3, Hras1, Nfatc2) that belonged to axon guidance pathway ordinarily found in neuronal cells. This is in line with the findings that HSCs express neural crest makers such as glial fibrillary acidic protein (GFAP), as well as neurotrophins and their receptors, which has suggested that HSCs might have a neural crest origin.

Our previous study demonstrated that HMGB1, a key DAMP molecule, may activate HSCs in a TLR4-MyD88-dependent manner by enhanced activities of downstream transcriptional factors NF-κВ and AP-1 and through the expression and secretion of the target gene MCP-1. The HMGB1 response is similar but weaker than effects elicited by LPS [23]. It has been unclear whether the TLR4 ligand effect of HMGB1 differs from that of exogenous TLR4 ligands on the gene expression networks of HSCs. In the present study, especially by the Venn analysis of the common and specific transcriptomic responses of HSCs to LPS or HMGB1 via TLR4 (i.e., the genes expression responsiveness only in JS1 but not in JS2), there are clearly TLR4-dependent and LPS-specific responsive genes that were enriched within pathways including Toll-like receptor, neurotrophin, MAPK, PI3K-Akt, and TNF. These signaling pathways typically respond to exogenous and endogenous ligands, cytokines, or hypoxia stress, and signal through MAPK, JNK, and/or NF-κB, to regulate the expression of genes with various functions including inflammation and immunity, homeostasis, cell-cycle control, metabolism, and oxidative stress resistance. On the other hand, the up-regulated TLR4-dependent and HMGB1-specific responsiveness genes were enriched within pathways of glutathione metabolism, metabolic, neurotrophin, and phagosome signaling, indicating a specific role of HMGB1 via TLR4 in provoking the system of cellular response to reactive oxygen intermediate and xenobiotics.

Our data indicate that there are differentially expressed genes that are TLR4-dependent and both LPS- and HMGB1-responsive. The common up-regulated genes were enriched to cell cycle, spliceosome, ribosome, and metabolism, which may reflect the active cellular responses to TLR4 ligands in wild-type HSCs, leading to extensive gene expression and protein synthesis and modification. Within the core regulatory genes identified, there were TRP53, Ccnd2, HK1, Ddx39b, and Ak2. These genes, especially TRP53, respond to diverse cellular stresses and regulate expression of target genes that are related to cell cycle arrest, apoptosis, senescence, DNA repair, or metabolism. In addition to the common up-regulated genes, the common TLR4-dependent down-regulated genes by LPS and HMGB1 were enriched within pathways including ubiquitin-mediated proteolysis, protein processing in endoplasmic reticulum, and MAPK signaling pathways. These may function in TLR4 downstream responses to either activate the downstream cascades or limit the responses as a feedback self-regulation, such as the down-regulation of Map3k1 and Prkca, which are the key components in MAPK signaling. These complex cascades illustrate how transcriptional regulation in HSCs is finely tuned and controlled by widely divergent regulatory pathways.

## Conclusions

The present study has demonstrated that TLR4 mediates an integrated signal transduction cascade linking many other important signaling pathways and function of HSCs. There are complex gene expression alterations subsequent to the loss of TLR4 in HSCs. Therefore, this signaling pathway regulates a wide spectrum of HSC functions, including inflammatory, fibrogenic, and chemotactic properties, as well as cell growth and metabolism. There are common and different regulatory signaling downstream of LPS and HMGB1 stimuli via TLR4 on HSCs. These findings emphasize the complex cascades downstream of TLR4 in the HSC that have significant consequences on its cell biology and function.

## Methods

### Cell treatment and RNA preparation

Immortalized wild-type (JS1) and TLR4^−/−^ (JS2) mouse HSC lines have been described in our previous study [22]. They were subcultured in 6-well plates (1 × 10^5^/ml per well) to 80 % confluence and divided into the negative control, LPS-, and HMGB1-treated groups. The cells were treated with phosphate buffer solution (PBS), 100 ng/ml LPS (purified lipopolysaccharides from *Escherichia coli* serotype 0111:B4, Sigma-Aldrich, St. Louis, MO, USA), or 100 ng/ml HMGB1 (Sigma), respectively, and collected at 24 h after treatment for RNA analysis. The cells were next treated with Trizol Reagent (Invitrogen, Carlsbad, CA, USA) and stored at −70 °C prior to RNA extraction. Total RNA extraction from three biological repeats of the cells was performed according to the manufacturer’s standard instructions (Invitrogen), and then, the RNA was prepared and purified using RNeasy Mini Kit (QIAGEN, Valencia, CA). RNA concentration was assessed by NanoDrop ND-1000 spectrophotometry (Thermo). High RNA quality was verified by formaldehyde denaturation electrophoresis.

### Microarray hybridization

The Agilent Array platform was employed for microarray analysis of the RNA samples. The sample preparation and microarray hybridization were performed based on the manufacturer’s standard protocols. Briefly, 1 μg of total RNA from each sample was amplified and transcribed into fluorescent cRNA with using the manufacturer’s Agilent’s Quick Amp Labeling protocol (version 5.7, Agilent Technologies). The labeled cRNAs were hybridized onto the Whole Mouse Genome Oligo Microarray (4x44K, Agilent Technologies). The arrays were scanned by the Agilent Scanner G2505B.

### Data analysis

Agilent Feature Extraction software (version 11.0.1.1) was used to analyze acquired array images. Quantile normalization and subsequent data processing were performed using the GeneSpring GX v11.5.1 software package (Agilent Technologies). Limma algorithm was used to screen the differentially expressed genes. The differentially expressed genes were selected according to the *P* value threshold (*P* < 0.05), followed by a secondary selection of >log1.5-fold difference.

### GO analysis

Fisher test was carried out for the analysis of significant gene ontology (GO-Analysis), which is a functional analysis associating differentially expressed genes with GO categories. The GO categories are derived from Gene Ontology (www.geneontology.org), which comprise three structured networks of defined terms that describe gene product attributes. The *P* value denotes the significance of GO term enrichment in the differentially expressed gene list. A *P* value ≤0.05 is considered to be significant. Fisher’s exact test was used to classify the GO category, and the false discovery rate (FDR) was calculated to correct the *P* value.

### Pathway analysis

Pathway analysis was used to identify biological pathways in which there is a significant enrichment of differentially expressed genes according to latest KEGG database. Fisher’s exact test followed by Benjamini–Hochberg (BH) multiple testing correction was calculated to select the significant pathway. Enrichment provides a measure of the significance of the function, and the threshold of significance was defined by *P* value and FDR.

### Genes-act-network analysis

The differential genes that populated significant pathways category were selected to build genes-act-network (Gene-Act-Net) according to the relationship between the genes, proteins, and compounds in the KEGG database. The flowchart of signaling transduction constructed by the differential genes and the central key regulatory genes were obtained by this analysis.

### Co-expression network analysis

The differential genes that populated both the significant pathways and GO category were selected to build gene co-expression networks according to the normalized signal intensity of specific expression genes. Pearson’s correlation was calculated for each pair of genes, and the significant correlation pairs were chosen to construct the network [39, 40] in both the control and case group. Core regulatory factors were determined by the degree (the link numbers one node has to the other) differences between the case and control networks [41, 42]. Moreover, K-cores in graph theory were introduced as a method of simplifying graph topology analysis. A K-core of a network is a subnetwork in which all nodes are connected to at least K other genes in the subnetwork [41, 43].

### Venn analysis

Venn analysis was used to analyze the common or differential TLR4-dependent genes expression in response to LPS or HMGB1 stimuli (Fig. 2). The differentially expressed genes that were categorized to be TLR4-dependent and LPS- or HMGB1-specific or common to both were further processed by GO and pathway analysis (Fig. 3) and build genes-act-network (Fig. 4).

### Quantitative RT-qPCR

JS1 and JS2 HSCs were stimulated with saline vehicle (control), 100 ng/ml LPS, or 100 ng/ml HMGB1 for 24 h as described above. Total RNA was extracted with TRIzol reagent (Generay Biotech, Shanghai, China) and then reverse transcribed to cDNA using a PrimeScriptTM RT reagent Kit (TaKaRa, Japan). Quantitative real-time PCR analysis (RT-qPCR) was performed using SYBR Green Realtime PCR Master Mix (Toyobo, Japan) on a 7500 Real-Time PCR Systems (Applied Bio systems, USA). The primer pairs were listed in Table 1. Data are represented as the fold changes of the expression level of the verified genes in JS1 cells relative to JS2 cells.

### Statistical analysis

Statistical differences were analyzed with ANOVA using SPSS software (17.0) (Chicago, IL, USA). The results were represented as mean ± standard deviation (SD), and the differences were considered statistically significant at *P* < 0.05.

## Additional files

Additional file 1: Table S1. A summary of transcriptomic analysis of JS1 and JS2 cells. (DOCX 62 kb) Additional file 2: Figure S1. Co-expression network analysis of JS1 (case) and JS2 cells (control) using differentially expressed genes that populated the pathways category. (TIF 15343 kb) Additional file 3: Figure S2. Gene-act-network analysis of the LPS response in TLR4 intact JS1 (A) and null JS2 (B) hepatic stellate cells. Green circles represented down-regulated genes; red circles represent the up-regulated genes; → activation/association; —: compound; —|: inhibition. The gene interaction network in TLR4 null cells post LPS stimulation were significantly simpler and lacked core regulatory factors. (TIF 3255 kb) Additional file 4: Figure S3. Co-expression network analysis of the LPS response in TLR4 intact JS1 (case) and null JS2 (control) hepatic stellate cells. (TIF 6926 kb) Additional file 5: Figure S4. Gene-act -network analysis of the HMGB1 response in TLR4 intact JS1 (A) and null JS2 (B) hepatic stellate cells. Green circles represented down regulated genes; red circles represent the up regulated genes; → activation/association; —: compound; —|: inhibition. The gene interaction network in TLR4 null cells post HMGB1 stimulation were significantly simpler and lacked core regulatory factors. (TIF 4371 kb) Additional file 6: Figure S5. Co-expression network analysis of the HMGB1 response in TLR4 intact JS1 (case) and null JS2 (control) hepatic stellate cells. (TIF 5866 kb)

## Abbreviations

DAMP: damage-associated molecular pattern molecule
HMGB1: high-mobility group box 1
HSC: hepatic stellate cells
KEGG: Kyoto Encyclopedia of Genes and Genomes
LPS: lipopolysaccharide
MAPK: mitogen-activated protein kinase
NF-κB: nuclear factor-κB
PI3K: phosphatidylinositol 3-kinase
TIMP: tissue inhibitor of metalloproteinase
TLR4: Toll-like receptor 4
α-SMA: α-smooth muscle actin
