# Supplementary material for: Transcriptomic analysis of the effects of Toll-like receptor 4 and its ligands on the gene expression network of hepatic stellate cells
Source: Fibrogenesis Tissue Repair. 2016 Feb 18;9:2. doi: 10.1186/s13069-016-0039-z (PMC4759739; doi:10.1186/s13069-016-0039-z)
Supplement: Additional file 1: Table S1. — A summary of transcriptomic analysis of JS1 and JS2 cells. (DOCX 62 kb) [file 13069_2016_39_MOESM1_ESM.docx]

**Supplemental Table 1. A summary of transcriptomic analysis of JS1 and JS2 cells**

| Groups | Total No. of Dif. genes | Change model | No. of Dif. genes | GO term | Pathway |
| --- | --- | --- | --- | --- | --- |
| JS1N *vs* JS2N | 5421 | up | 2748 | 102 | 17 |
|  |  | down | 2673 | 67 | 10 |
| JS1L *vs* JS1N | 1392 | up | 849 | 69 | 10 |
|  |  | down | 543 | 86 | 8 |
| JS2L *vs* JS2N | 1431 | up | 477 | 55 | 1 |
|  |  | down | 954 | 62 | 8 |
| JS1H *vs* JS1N | 1445 | up | 586 | 47 | 8 |
|  |  | down | 859 | 95 | 5 |
| JS2H *vs* JS2N | 1253 | up | 503 | 42 | 5 |
|  |  | down | 750 | 63 | 7 |

JS1N: TLR4 wild type mouse stellate cells (JS1) treated with normal saline group; JS2N: TLR4-/- mouse stellate cells (JS2) treated with normal saline group;

JS1L: TLR4 wild type mouse stellate cells (JS1) treated with LPS group; JS2L: TLR4-/- mouse stellate cells (JS2) treated with LPS group; JS1H: TLR4 wild type mouse stellate cells (JS1) treated with HMGB1; JS2H: TLR4-/- wild type mouse stellate cells (JS2) treated with HMGB1.
