# Supplementary figures and images for: Transcriptomic analysis of the effects of Toll-like receptor 4 and its ligands on the gene expression network of hepatic stellate cells
Source: Fibrogenesis Tissue Repair. 2016 Feb 18;9:2. doi: 10.1186/s13069-016-0039-z (PMC4759739; doi:10.1186/s13069-016-0039-z)

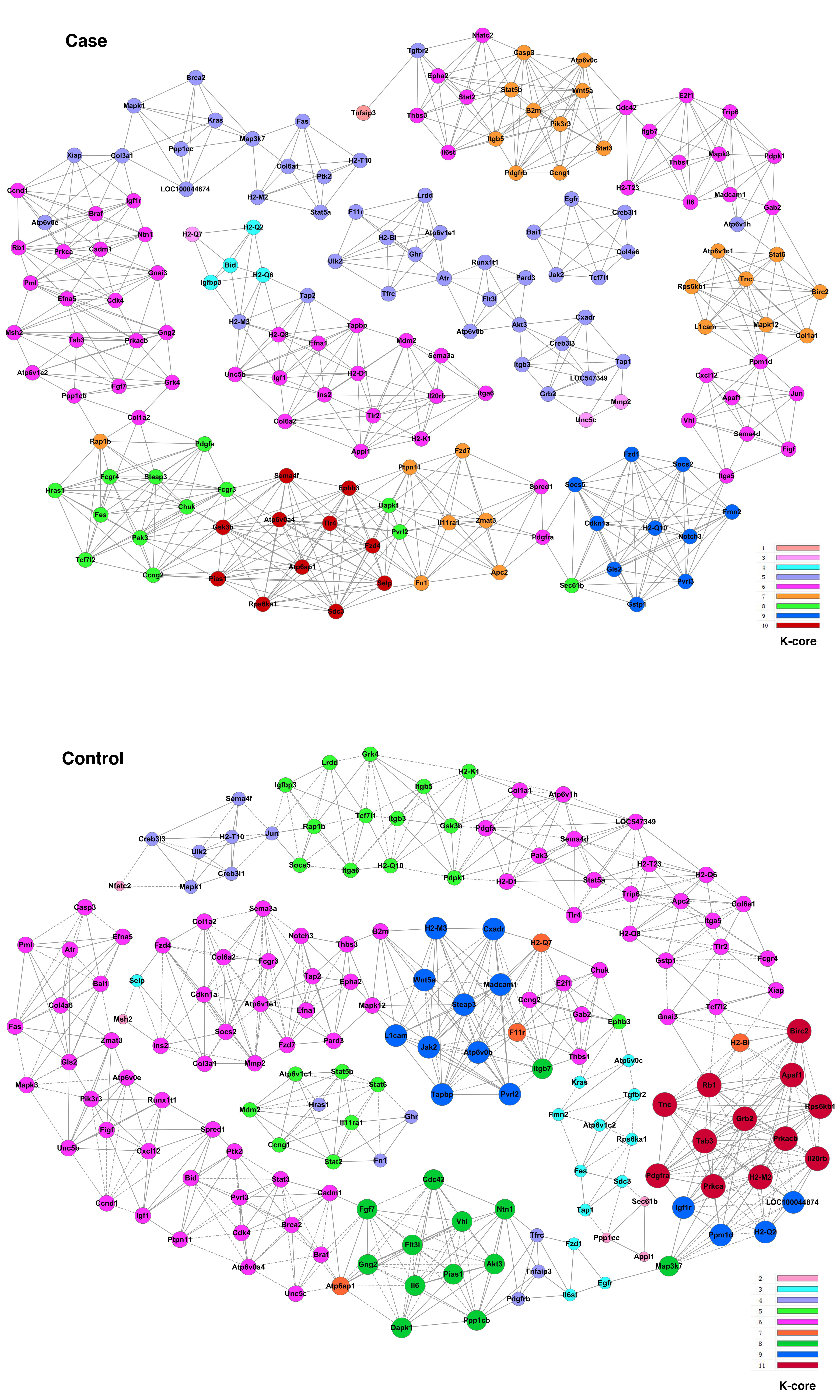

Supplement: Additional file 2: Figure S1. — Co-expression network analysis of JS1 (case) and JS2 cells (control) using differentially expressed genes that populated the pathways category. (TIF 15343 kb) [file 13069_2016_39_MOESM2_ESM.tif]

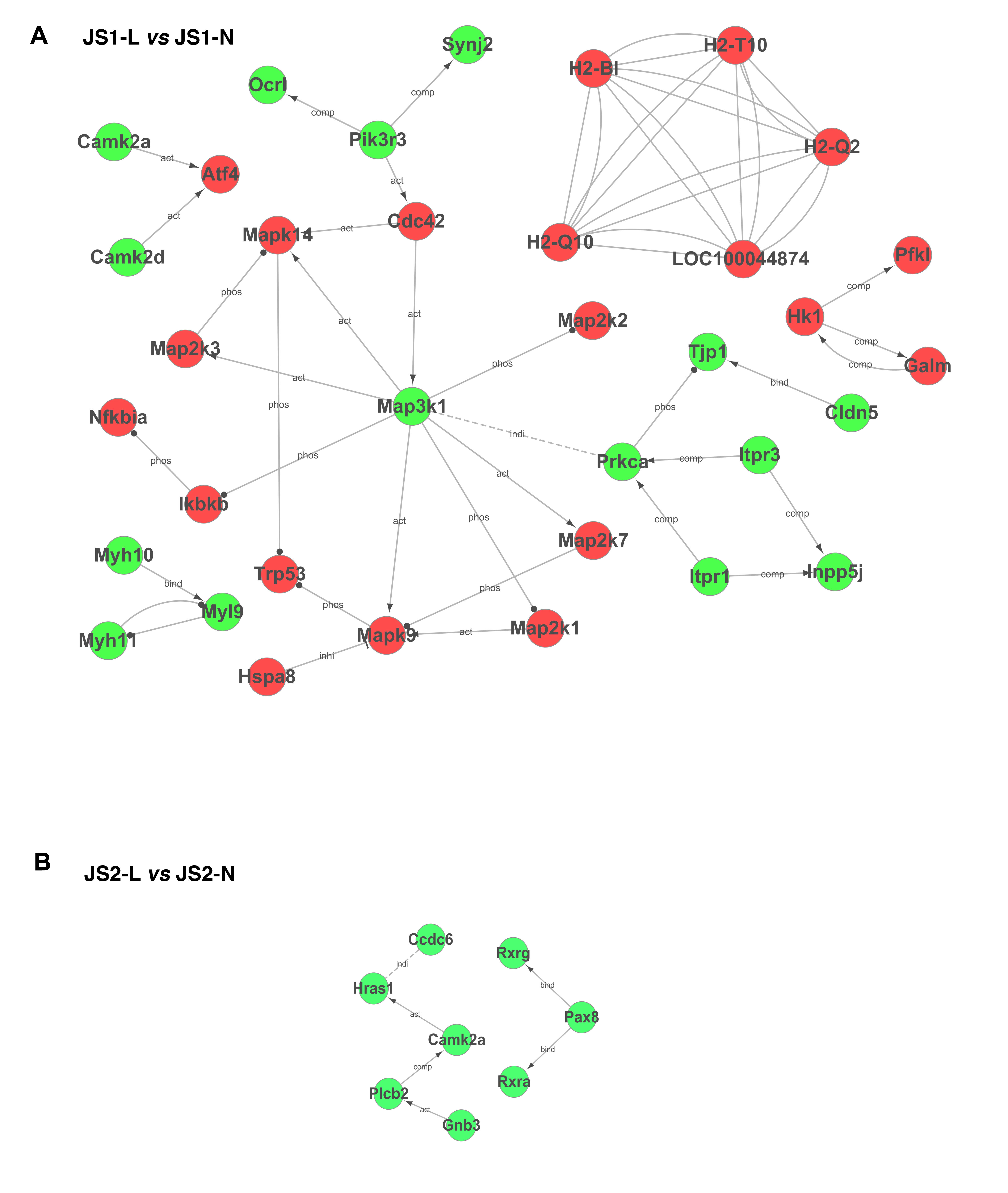

Supplement: Additional file 3: Figure S2. — Gene-act-network analysis of the LPS response in TLR4 intact JS1 (A) and null JS2 (B) hepatic stellate cells. Green circles represented down-regulated genes; red circles represent the up-regulated genes; → activation/association; —: compound; —|: inhibition. The gene interaction network in TLR4 null cells post LPS stimulation were significantly simpler and lacked core regulatory factors. (TIF 3255 kb) [file 13069_2016_39_MOESM3_ESM.tif]

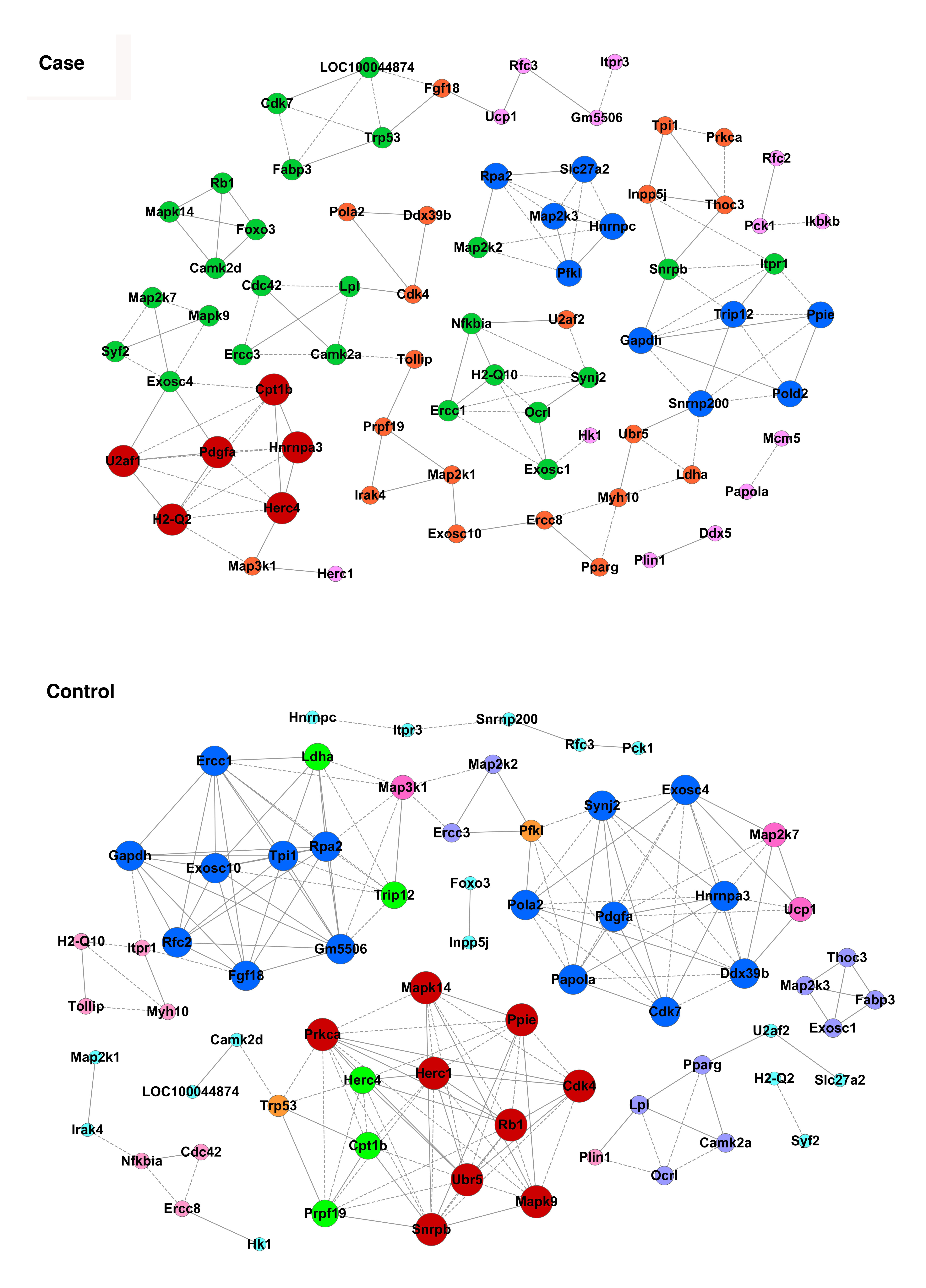

Supplement: Additional file 4: Figure S3. — Co-expression network analysis of the LPS response in TLR4 intact JS1 (case) and null JS2 (control) hepatic stellate cells. (TIF 6926 kb) [file 13069_2016_39_MOESM4_ESM.tif]

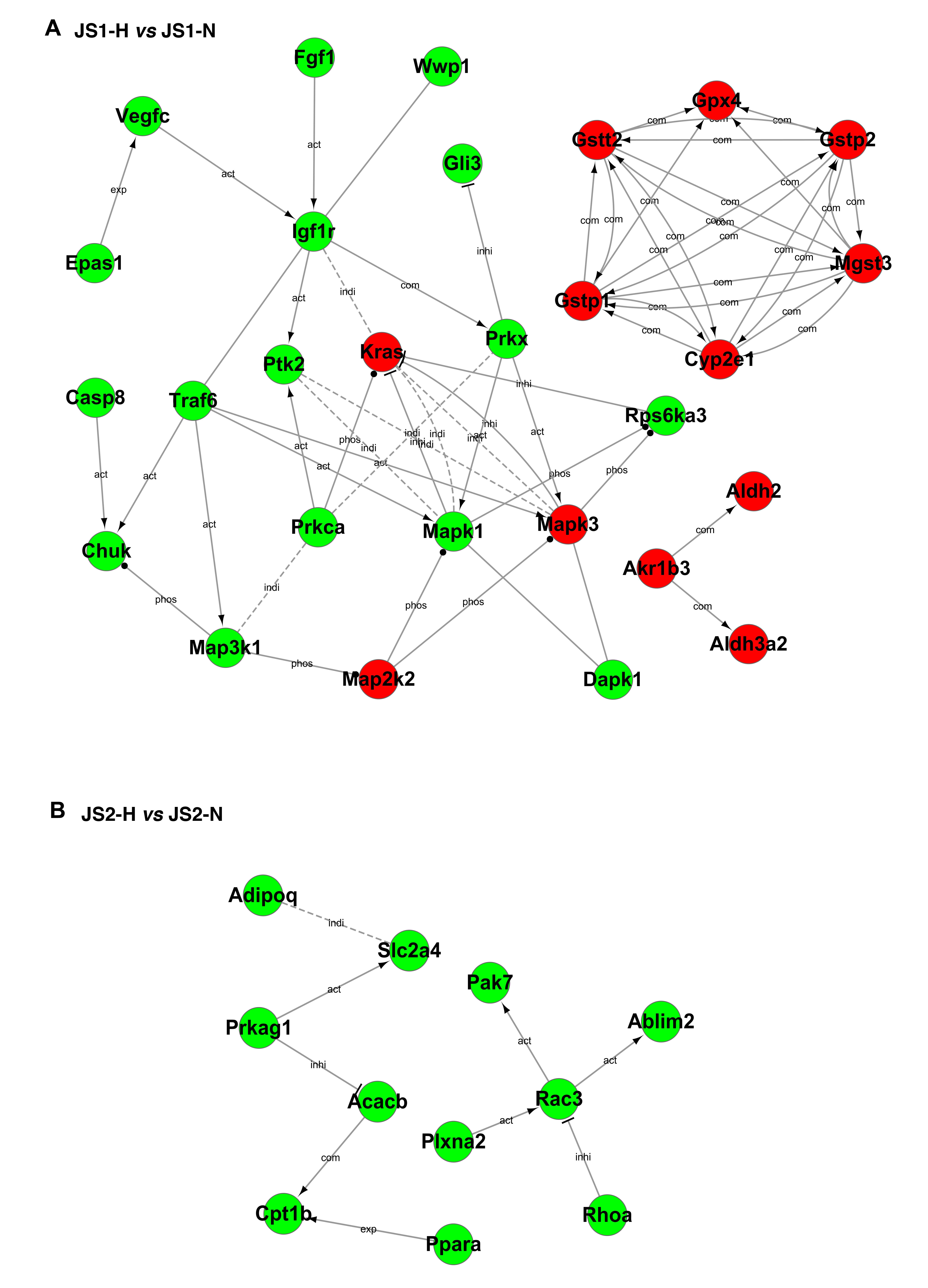

Supplement: Additional file 5: Figure S4. — Gene-act -network analysis of the HMGB1 response in TLR4 intact JS1 (A) and null JS2 (B) hepatic stellate cells. Green circles represented down regulated genes; red circles represent the up regulated genes; → activation/association; —: compound; —|: inhibition. The gene interaction network in TLR4 null cells post HMGB1 stimulation were significantly simpler and lacked core regulatory factors. (TIF 4371 kb) [file 13069_2016_39_MOESM5_ESM.tif]

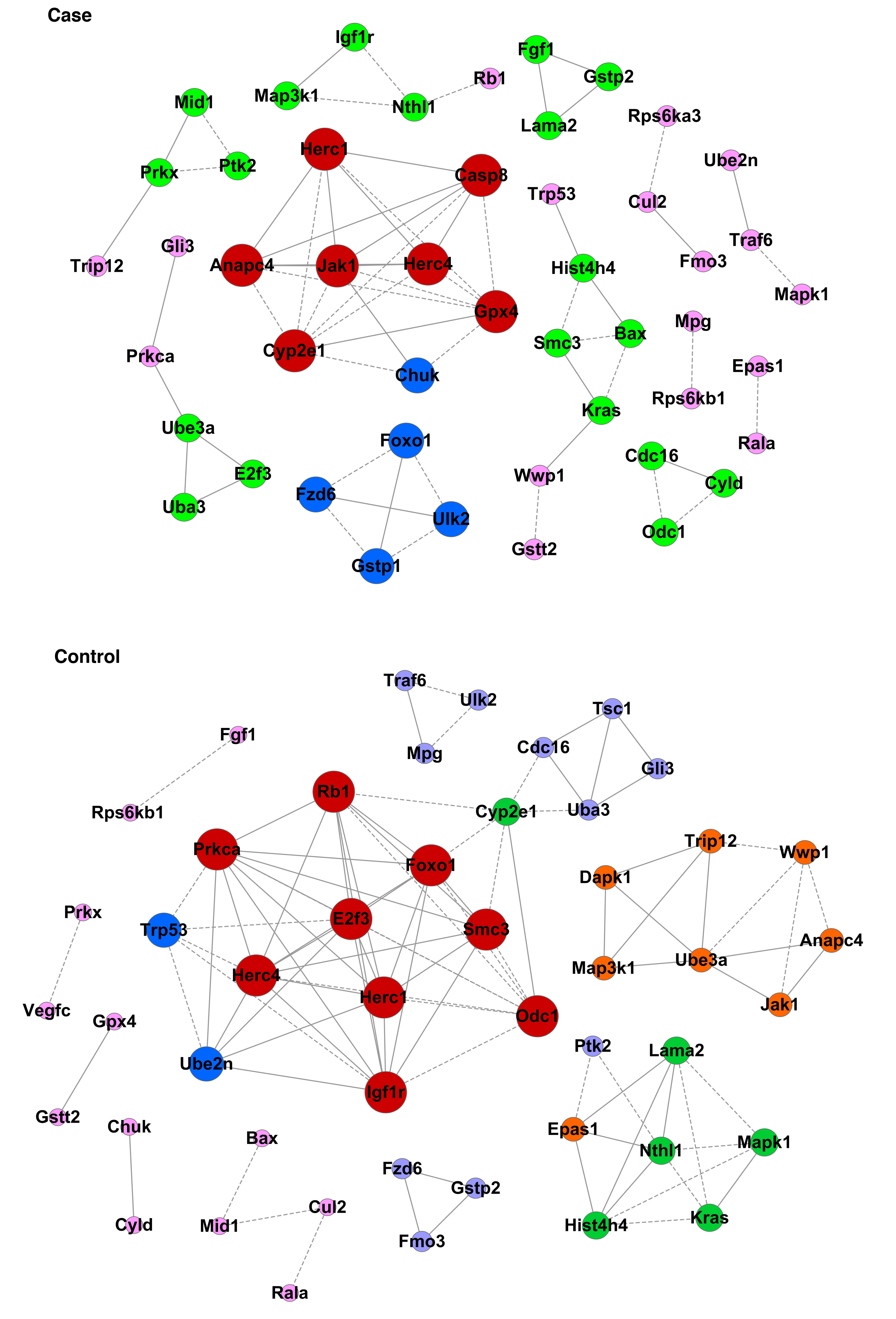

Supplement: Additional file 6: Figure S5. — Co-expression network analysis of the HMGB1 response in TLR4 intact JS1 (case) and null JS2 (control) hepatic stellate cells. (TIF 5866 kb) [file 13069_2016_39_MOESM6_ESM.tif]
